# Social anxiety and attentional bias to negative emotional information: the relationship and intervention

**DOI:** 10.1186/s12888-024-05938-2

**Published:** 2024-07-17

**Authors:** Chen Liu, Jon D. Elhai, Christian Montag, Haibo Yang

**Affiliations:** 1 Academy of Psychology and Behavior, Faculty of Psychology, Tianjin Normal University, Xiqing District, No. 393 Binshuixi Road, Tianjin, 300387 China; 2 Department of Psychology, University of Toledo, Toledo, Ohio 43606 USA; 3 Department of Psychiatry, University of Toledo, Toledo, Ohio 43614 USA; 4 Department of Molecular Psychology, Institute of Psychology and Education, Ulm University, 89081 Ulm, Germany; 5 Tianjin Social Science Laboratory of Students’ Mental Development and Learning, Tianjin, 300387 China; 6 Academy of Mechnanical Engineering, Tianjin Sino-German University of Applied Sciences, No. 2 Yashen Road, Jinnan District, Tianjin, 300350 China

**Keywords:** Social anxiety, Attentional bias modification, Negative emotional information, Dot-probe tasks

## Abstract

**Background:**

According to the cognitive behavioral model of social anxiety, attentional bias to negative emotional information causes and maintains anxiety. The goal of attentional bias modification (ABM) is to reduce anxiety by reducing attention bias to negative emotional information.

**Method:**

We used questionnaires and experiments to explore the improvement effect of ABM training on social anxiety in college students. In Study 1, we used dot-probe tasks to investigate the attentional bias to negative emotional information and the relationship with social anxiety severity in college students. In Study 2, college students with high social anxiety were divided into two groups: attentional bias modification training task group (ABM) and attention control condition task group (ACC). The ABM group received a continuous intervention for 10 days to observe changes in social anxiety levels and attentional bias scores in the pretest and posttest stages.

**Results:**

The results showed that the correlation of attentional bias to negative emotional information and social anxiety severity was significant. Meanwhile, the high social anxiety participants responded more quickly to negative emotional information. After the intervention, social anxiety levels and attentional bias scores of the training group were significantly reduced.

**Conclusions:**

The results showed that attentional bias modification training can reduce attentional bias to negative emotional information in college students with social anxiety and effectively improve their social anxiety.

**Supplementary Information:**

The online version contains supplementary material available at 10.1186/s12888-024-05938-2.

## Introduction

Social anxiety disorder, also known as social phobia, refers to anxiety and fear caused by the possibility of being evaluated and scrutinized when individuals are in contact with others [1, 2]. Studies have found that social anxiety often appears in college students [1, 3]. Long-term social anxiety can greatly affect learning, life and other functional aspects, which is harmful to physical and mental health. However, compared with other mental disorders, social anxiety disorder is not easy to detect and ignore. Eventually, extreme anxiety and depression can easily occur due to a lack of early intervention [4, 5].

The cognitive behavioral model of social anxiety points out that individuals with social anxiety are more sensitive to negative emotional information and may have attentional bias [6–9], and the maintenance of an anxiety state may be caused by attentional bias to negative emotional information [10]. Individuals with social anxiety have a certain degree of attentional bias to negative information, which is an important process of anxiety generation [11]. That can be understood as the allocation of attention to negative stimuli being different from that to neutral stimuli [11]. Therefore, the attentional bias to negative emotional information may be significantly correlated with social anxiety severity. If the attention bias of individuals with social anxiety is improved, their social anxiety level may be alleviate. However, some studies have not found the attentional bias to negative emotional information [12, 13]. The relationship between social anxiety and the attentional bias to negative emotional information still needs further investigation.

The attentional vigilance-avoidance hypothesis indicates that individuals with anxiety involve entering the stage of automatic capture of negative information, followed by an increase in anxiety, and immediately entering the avoidance stage to reduce the anxiety experience [14]. Therefore, attentional bias to negative information may be intervened to achieve the goal of alleviating social anxiety.

As we all know, there are many intervention methods for social anxiety from the cognitive-behavioral perspective, and attentional bias modification training (ABM) is one of the common methods. This method is a computerized self-help attentional bias treatment that aims to reduce anxiety levels by manipulating individuals’ attentional bias to negative stimuli [15].

There have been many intervention studies on ABM for anxiety and for other targets, but the results are mixed. The changes in the range of attentional bias scores of training group participants was larger than that in the control group after 10 consecutive days of ABM [16]. A recent meta-analysis showed that training can significantly reduce anxiety symptoms and attentional bias to threat stimuli and indicated that the greater the total number of trials, the greater the efficacy [17].

Although ABM can achieve intervention effects, some studies have not found significant differences when compared to control groups [18–21]. Some studies used ABM twice a week for 3 weeks and found no significant difference in the pretest and posttest stages in the training group [22]. The reasons for these different results are manifold. First, different studies used different experimental materials. For example, the negative materials only involved aversive stimuli, and further research is needed to determine whether the treatment effect is specific to aversive stimuli or can be generalized to other types [21]. The study used foreign emotional faces, and cultural differences may affect individual recognition of facial emotions [19]. Second, there were differences in training procedures and environments among the studies. For example, a study adopted internet-based attention training in the home environment, which may affect the conscientiousness of individuals in training [20]. Finally, there were differences in training time intervals and frequency. For example, a study assessed participants only twice a week, and thus the time interval was too long [22]. As a result, no significant difference was found in anxiety levels of the training group in the pretest and posttest stages. A study did not find significant intergroup differences in social anxiety symptoms after five consecutive days of intervention [19]. There have been differences in training settings in the past, the training times of many studies were too short [19, 20], and the training intervals were too long [22, 23], which may be the reason for previous mixed results. Therefore, it is necessary to further optimize experimental materials, training procedures and training settings in future studies.

Based on that information, this study used the Standardized Chinese Facial Emotion Picture System (CFAPS) [24] to conduct attentional bias training for 10 consecutive days, and it was assumed that attentional bias training would have a better effect on social anxiety under intensive training conditions [16].

Considering the above theories and previous research results, we proposed the following hypotheses. Hypothesis H1: The attentional bias to negative emotional information is significantly correlated with the Social Interaction Anxiety Scale (SIAS) and the Social Phobia Scale (SPS) scores. Hypothesis H2: Individuals with high social anxiety show attentional bias to negative emotional information. Hypothesis H3: In the posttest stage, social anxiety levels and attentional bias scores of the ABM (Attentional bias modification training task) group is significantly lower than those of the ACC (Attention control condition task) group. Hypothesis H4: In the posttest stage, social anxiety levels and attentional bias scores of the ABM group decrease significantly.

## Experiment 1

We explored the correlation of attentional bias for negative emotional information and social anxiety severity in individuals. The study hypothesized that the attentional bias to negative emotional information is significantly correlated with SIAS and SPS scores.

### Materials and methods

#### Participants

We recruited 67 participants (female = 26; male = 41) from colleges and universities, in the form of issuing second-class activities which is a social practice outside the classroom compared with classroom teaching. College students have certain credit requirements for the second-class activities when they graduate. The age range of the participants was 17–22 years old. All the participants were right-handed and had no mental illness. Informed consent was signed before the experiment, and compensation was given after the experiment.

#### Measures

The Social Interaction Anxiety Scale (SIAS) has 19 items and uses 5-point scoring to measure anxiety and fear in the context of communication with people [25]. The higher the score, the higher the degree of social anxiety. The SIAS scale has been widely used in the study of social anxiety among college students and has been proven to have good representativeness [26]. The average of norm data for Chinese college students is 49.10. The revised Chinese version had a Cronbach’s α coefficient of 0.874 [25]. In this study, Cronbach’s α coefficient was 0.90.

The Social Phobia Scale (SPS) has a total of 20 items [25] and uses 5-point scoring to measure anxiety and fear in performance/observed situations. The SPS scale has good validity in social anxiety studies [27, 28]. The revised Chinese version had a Cronbach’s α coefficient of 0.904 [25]. In this study, Cronbach’s α coefficient was 0.92.

#### Materials and procedure

At present, most studies on attentional bias use stimulus materials such as words or pictures. However, the words mainly depend on their symbolic meaning and need to be processed by human speech system, with relatively low stimulation and ecological validity [29, 30]. Therefore, this study uses emotional pictures (CFAPS) that are more intuitive and can measure the initial reactions. In addition, to ensure the effectiveness of the experimental materials, 30 participants (15 males and 15 females, with an average age of 18.47 ± 0.68 years) who did not participate in the formal experiment were selected to give a 9-point score on the potency, arousal and familiarity of each picture [31]. Finally, 40 emotional faces were selected, including 20 negative and neutral emotional faces, half for men and half for women. The results of paired sample *t*-test analyses showed no significant differences in familiarity or arousal between the two types of materials, but a significant difference in potency (*t* = 8.03, *P* < 0.001, *d* = 1.47) (Table 1). The picture size was 260 × 300. The program ran E-Prime, version 2 (Psychology Software Tools, Pittsburgh).

**Table 1:** Assessment of picture materials [*M* ± *SD*]

Variable	Negative emotional face	Neutral emotional face
Potency	2.92 ± 0.98	3.51 ± 1.05
Arousal	3.99 ± 1.36	4.10 ± 1.30
Familiarity	4.19 ± 1.63	4.23 ± 1.58

*M* = Mean, *SD* = standard deviation

The study adopted a 2 (Emotional type: negative and neutral) × 2 (Location of probe: consistent and inconsistent) within-subjects experimental design. The emotional type and location of the probe were the within-subjects variables, and the dependent variables were attentional bias scores of negative emotional face. Among the probes, “consistent” meant that the probe was behind the negative emotional face, and “inconsistent” meant that the probe was behind the neutral emotional face.

The study was conducted on a 14-inch desktop computer monitor. Before the experiment, participants were informed of the experimental content and operation process. The experimental procedures for each participant were as follows: practice experiment (8 trials) and formal experiment (72 trials). The emotional faces in the practice experiment do not appear in the formal experiment. After that, participants completed the SIAS, SPS.

**Fig. 1:**
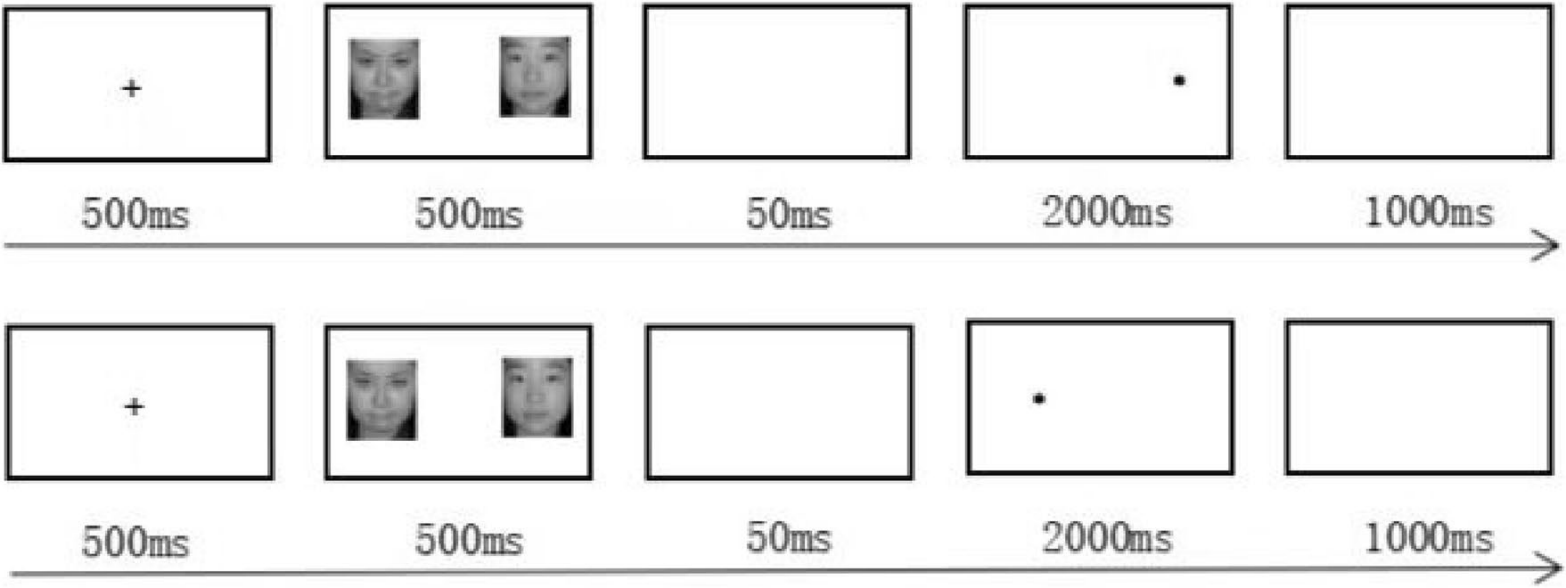
Flowchart of the dot-probe task paradigm

Figure 1 illustrates the experimental flow. First, participants focused on the cross-fixation point “ + ” in the center of the screen. After 500 ms, a pair of negative and neutral emotional faces appeared on the left and right sides of the screen for 500 ms with a 50% probability each, and then a blank screen appeared for 50 ms. A probe (black “●”) randomly appeared in the center of any previous face with a 50% probability. The probe pressed the “F” key and the “J” key at the left and right positions, respectively. The participants needed to respond to the key quickly and accurately within 2000 ms, with an interval of 1000 ms. The participants completed the practice experiment first and entered the formal experiment after all reactions were correct.

#### Data analysis

The reaction times less than 200 ms are too fast to be considered a cognitive process and reaction times more than 1200 ms are too slow and can be difficult to conclude whether it was due to lack of attention, fatigue or other reasoning other than the cognitive processes [32]. Therefore, data with reaction times of less than 200 ms and more than 1200 ms were deleted, meanwhile, invalid trials with incorrect key reactions were deleted. The specific demographic information is shown in Table 2.

**Table 2:** Means, standard deviations and Pearson correlations (*n* = 67)[*M* ± *SD*]

Variable	M ± SD	1	2	3
SIAS	39.88 ± 11.53	1		
SPS	37.34 ± 13.08	0.823***	1	
Attentional bias scores	-1.50 ± 9.55	0.353**(0.003)	0.305*(0.012)	1
age(year)	19.13 ± 1.01			
gender	67(26)			

*M* = Mean, *SD* = standard deviation; *P < 0.05, **P < 0.01, ***P < 0.001, *SIAS* Social Interaction Anxiety Scale, *SPS *Social Phobia Scale, Numbers in gender brackets are female

In the negative-neutral emotional face pairs, the difference between the reaction time under inconsistent conditions and that under consistent conditions was the attentional bias score. If attentional bias scores were positive, it indicated that the participant reacted faster to negative emotional faces. If attentional bias scores were negative, it indicated that the participant reacted faster to neutral emotional faces. Therefore, the higher the score, the faster the participant reacted to negative emotional faces. In this study, the correct response rate of the effective participants was 100%, which was not meaningful for comparison. The experimental data were analyzed by SPSS 19.0.

### Results

As shown in Table 2, the Pearson correlations results showed that attentional bias is significantly correlated with SIAS (*r* = 0.353, *p* = 0.003) and SPS (*r* = 0.305, *p* = 0.012), while the correlation between SIAS and SPS is significant (*r* = 0.823, *p* < 0.001).

### Discussion

In Experiment 1, behavioral experiments and questionnaires were used to study the correlations between social anxiety and attentional bias to negative emotional information. The results showed that.

attentional bias to negative emotional information is significantly correlated with SIAS and SPS scores. According to the Orientation Theory of Attentional bias, socially anxious individuals have attentional bias to negative emotional faces [16, 33]. Therefore, hypothesis 1 was verified.

Previous study showed that the higher the level of social anxiety, the more attentional bias college students showed to negative emotional information [34]. The meta-analysis on attentional bias and anxiety disorders and found that participants in the anxiety group had greater attentional bias [15]. The attentional bias of socially anxious individuals to negative stimuli was the key reason for their anxiety state to be maintained [6]. Other studies found that individuals with social difficulties were more sensitive to negative emotional stimuli and might have had attentional bias [9]. Thus, we asked if we could reduce levels of social anxiety through attentional bias training to achieve the purpose of improving social anxiety. Therefore, study 2 was carried out.

## Experiment 2

Based on study 1, we explored the intervention effect of ABM on social anxiety of college students.

### Methods

#### Participants and measures

We recruited 527 participants from a university in Tianjin through a convenient sampling method. Participants were invited to fill an online questionnaire. Finally, we obtained a total of 404 effective questionnaires excluding those with a response time of less than 600 s and more than 2100s considering the authenticity of the reaction and those who had not completed the questionnaire. SIAS scores were ranked from high to low, and the latter 25% of participants were placed in the low social anxiety group [35]. The purpose was to verify attentional bias characteristics of individuals with social anxiety to negative emotional information, and 31 participants in the low social anxiety group participated in the experiment. The top 25% of participants were in the high social anxiety group [35]. The lowest SIAS score for the high social anxiety group is 47, which is close to the average of the norm data for Chinese university students, therefore the grouping of the high social anxiety group was effective. Finally, 62 participants in the high social anxiety group participated in the whole experiment. The post-hoc power analysis demonstrated that the power (1-β) was 0.97, which meant the number of subjects was sufficient. The participants were randomly divided into two groups. Neither the researchers nor participants knew about the group assignments. All participants were right-handed and had no mental illness. The informed consent form was signed before the experiment, and the second-class activity credits and learning supplies were given in return after the experiment. All the measures are same to study 1. The specific demographic information of the two groups is shown in Table 5.

#### Materials and Procedure

According to the method of study 1, 76 negative and neutral emotional faces (half male and half female) were finally selected, as shown in Table 3. Twenty-five pairs of negative-neutral face pairs were formed, of which 9 pairs were used in the attentional bias measurement task, and 8 pairs of materials were used in the ABM and ACC tasks; 13 pairs of neutral–neutral face pairs were formed, and 5 pairs were used in the attentional bias measurement task, attentional bias training task and attention control task. Paired samples *t*-tests were used to analyze the potency, arousal and familiarity of the pictures. The results showed no significant differences in familiarity or arousal between the two kinds of materials, but a difference in potency (*t* = 10.64, *P* < 0.001, *d* = 1.94) was significant.

**Table 3:** Assessment of picture materials [*M* ± *SD*]

Variable	Negative emotional face	Neutral emotional face
Potency	2.53 ± 1.02	4.09 ± 1.04
Arousal	4.16 ± 1.70	3.94 ± 1.21
Familiarity	4.14 ± 1.82	4.32 ± 1.49

*M* = Mean, *SD* = standard deviation

**Fig. 2:**
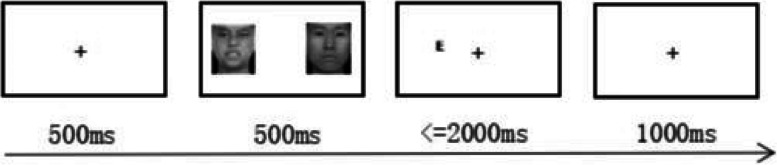
Flowchart of the attentional bias measurement task

According to the intervention parameters used in previous studies [13, 16], to further reduce any material effects that led to training effects, a research paradigm different from Study 1 was used in the experiment. The study is divided into the attentional bias measurement task, ABM and ACC task. See Fig. 2 for the flowchart of the attentional bias measurement task:

#### Design and Task

The study adopted a 2 (Group: ABM group and ACC group) × 2 (Measurement time: pretest and posttest) mixed experimental design. The between-groups variable was the group, and the within-subjects variable was measurement time. The dependent variables included attentional bias scores and scores of each symptom assessment questionnaire. On the first day of the formal experiment, all participants needed to complete the attentional bias measurement task (pretest) first and then received different treatments according to different groups. The participants were trained once a day for a total of 10 days. On the last day of training, participants completed their training tasks first, then completed the attentional bias measurement task (posttest), and completed the SIAS, SPS questionnaires.

1. Attentional bias measurement task (pretest and posttest)

In the attentional bias measurement task, a cross-fixation point “ + ” appeared in the center of the screen for 500 ms, and participants were required to look directly at the fixation point. After the fixation point disappeared, an emotional face picture was presented on the left and right sides. The emotional pictures in Study 2 were from the same source as those in Study 1, but they were different pictures. A total of 28 (9 negative emotional faces and 19 neutral emotional faces) faces were selected and presented for 500 ms. Then, the screen would display the reaction target, i.e., “E” or “F” (the probability of occurrence of the two letters was the same) at any position where two pictures had appeared. When the letter “E” appeared, participants were instructed to press the “E” key on the keyboard, and when the letter “F” appeared, press the “F” key on the keyboard. The participants were required to respond quickly and accurately within 2000 ms, and there were 1000 ms empty screens between different trial times. The task included 16 trials of the practice experiment and 160 trials of the formal experiment, of which 128 trials were negative-neutral face pairs, and the remaining 32 trials were filled trials, presenting neutral–neutral face pairs [36]. When the accuracy rate of the practice experiment reached 100%, it automatically entered the formal experiment, which took approximately 8 min.

(2) Attentional bias modification training (ABM) task

The ABM task was similar to the attentional bias measurement task. Another 48 pictures were selected and randomly divided into 2 groups. Each group included 16 neutral and 8 negative pictures, of which 8 neutral pictures were used as filling materials. The ABM task used one of these pictures. When the negative-neutral face pairs were presented (80%), the letter “E” or “F” consistently appeared after the neutral face (80%). Each task consisted of four blocks, with 160 trials in each block, for a total of 640 trials. There was a short break between each set of two blocks, which took approximately 20 min.

(3) Attention control condition (ACC) task

The ACC task was the same as the attentional bias measurement task, and another 24 pictures were selected. When the negative-neutral face pairs were presented (80%), the probe randomly appeared after the negative or neutral face with the same probability (40%). Each training session included four blocks, with 160 trials in each block, for a total of 640 trials. There was a short break between each set of two blocks, which took approximately 20 min.

#### Data analysis

The SIAS, SPS and attentional bias scores were used as dependent variables, and 2 (Group: ABM group and ACC group) × 2 (Measurement time: pretest and posttest) repeated-measures ANOVA was used. Independent samples *t*-tests were used to analyze the pretest and posttest of the two groups. A paired samples *t*-test was used to analyze the pretest and posttest results in each group. *P* < 0.05 indicated statistically significant differences between comparison groups.

### Results

#### Reaction time

As shown in Table 4, reaction time was first used as the dependent variable to perform repeated-measures ANOVA of a 2 (Group: low social anxiety group and high social anxiety group) × 2 (Location of probe: consistent and inconsistent) design. The main effect of group was significant: *F* (1, 91) = 7.95, *P* = 0.006, η^2^ = 0.08; the interaction was also significant: *F* (1, 91) = 4.01, *P* = 0.048, η^2^ = 0.04. The simple effect test showed that under consistent conditions, reaction time of the high social anxiety group was significantly lower than that of the low social anxiety group (*P* = 0.004). The high social anxiety group responded to negative emotional information faster than the low social anxiety group.

**Table 4:** Reaction time of the high and low social anxiety groups at different probes [*M* ± *SD*]

	Group	
Probe location	High social anxiety group (*n* = 62)	Low social anxiety group (*n* = 31)
Consistent	506.99 ± 71.22	551.62 ± 61.64
Inconsistent	508.65 ± 70.32	546.99 ± 57.42

*M* = Mean, *SD* = standard deviation

#### Questionnaires and attentional bias scores

The attentional bias, SIAS and SPS scores of each group are shown in Table 5.

**Table 5:** Attentional bias, SIAS and SPS scores [*M* ± *SD*]

	Group		*t*	*p*	Cohen’s d
Variable	High social anxiety group	Low social anxiety group			
Attentional bias scores	1.66 ± 15.27	-4.63 ± 11.98	-2.00*	0.048	0.46
SIAS	59.87 ± 8.46	26.03 ± 1.66	-30.36***	< 0.001	5.55
SPS	56.39 ± 11.77	23.26 ± 4.25	-19.74***	< 0.001	3.74
age	18.90 ± 0.97	19.13 ± 0.85	1.10	0.273	
gender	62(17)	31(6)			

*M* = Mean, *SD* = standard deviation, *P < 0.05, **P < 0.01, ***P < 0.001, *SIAS* Social Interaction Anxiety Scale, *SPS* Social Phobia Scale; Numbers in gender brackets are female

Using an independent samples *t*-test, the results showed that attentional bias scores of the high social anxiety group were significantly higher than those of the low social anxiety group (*t* = -2.00,* P* = 0.048, *d* = 0.46). Individuals with high social anxiety showed attentional bias to negative emotional information, and hypothesis 2 was verified.

#### Questionnaires and attentional bias scores in the ABM and ACC groups

Table 6 shows scores of the self-report questionnaires and attentional bias in the pretest and posttest of each group.

**Table 6:** Comparison of self-report questionnaire scores and attentional bias in the pretest and posttest [*M* ± *SD*]

Variable	pretest and posttest	ABM group (*n* = 31)	ACC group (*n* = 31)
SIAS	pretest	58.23 ± 5.05	61.52 ± 10.69
	posttest	46.55 ± 9.59	54.23 ± 15.02
SPS	pretest	56.52 ± 11.44	56.26 ± 12.27
	posttest	41.87 ± 12.35	49.94 ± 14.91
Attentional bias scores	pretest	2.08 ± 13.00	1.24 ± 17.46
	posttest	-5.21 ± 14.90	3.00 ± 14.19

*M* = Mean, *SD* = standard deviation

With SIAS as the dependent variable, 2 (Group: ABM group and ACC group) × 2 (Measurement time: pretest and posttest) repeated-measures ANOVA showed that the group main effect was significant: *F* (1, 60) = 5.55, *P* = 0.022, η^2^ = 0.09; the main effect of time was significant: *F* (1, 60) = 45.87, *P* < 0.001, η^2^ = 0.43. With SPS as the dependent variable, 2 (Group: ABM group and ACC group) × 2 (Measurement time: pretest and posttest) repeated-measures ANOVA showed that the interaction was significant: *F* (1, 60) = 5.15, *P* = 0.027, η^2^ = 0.08. The simple effect test showed that the SPS scores in the ABM group were significantly lower than those in the ACC group at the posttest stage (*P* = 0.024), and hypothesis 3 was partially verified. The SPS scores of the ABM group in the pretest and posttest stages were significantly different (*P* < 0.001), and the posttest scores were significantly lower than the pretest scores. The SPS scores of the ACC group in the pretest and posttest stages were significantly different (*P* = 0.018). It can be seen from the difference in effect sizes that SPS scores of the ABM group in the pretest and posttest stages were greater.

With attentional bias scores of the ABM group and ACC group as the dependent variable, the independent samples *t*-test showed that before training, there was no significant difference between the ABM group and the ACC group (*t* = 0.21, *p* = 0.831). In the posttest stage, the ABM group’s attentional bias scores were significantly lower than those of the ACC group (*t* = -2.22, *p* = 0.030, *d* = -0.56). Therefore, hypothesis 3 was fully verified.

As shown in Table 6, the *t*-test results of paired samples showed that there was a significant difference in SIAS (*t* = 7.07, *P* < 0.001, *d* = 1.27), SPS (*t* = 6.07, *P* < 0.001, *d* = 1.09), and attentional bias scores (*t* = 2.24, *P* = 0.033, *d* = 0.40) of the ABM group in the pretest and posttest stages, and the scores in the posttest stage were significantly lower than those before training. The SIAS (*t* = 3.22, *P* = 0.003, *d* = 0.58) and SPS (*t* = 2.29, *P* = 0.029, *d* = 0.41) scores of the ACC group in the pretest and posttest stages were significantly different, and scores in the posttest stage were significantly lower than those before training. The effect size was moderate, and the difference in attentional bias scores was not significant. Moreover, the difference in SIAS and SPS scores in the ABM group during the pretest and posttest stages was greater than that in the ACC group. Finally, hypothesis 4 was verified.

### Discussion

In this study, behavioral experiments and questionnaires were used to explore the intervention effect of ABM on social anxiety. First, it was consistent with the result of study 1, that is, college students with high social anxiety showed attentional bias to negative stimuli. Second, study 2 found that ABM could reduce the attentional bias of socially anxious college students to negative emotional faces in dot-probe tasks, which was consistent with previous research results [37]. Through analysis, ABM could significantly reduce the level of social anxiety and showed a better intervention effect than ACC, which was consistent with the results obtained in previous studies [16, 23, 38, 39].

## General discussion

Through questionnaires and experiments, Study 1 showed that the attentional bias to negative emotional information is significantly correlated with SIAS and SPS scores. This was consistent with previous findings that, for individuals with social anxiety, alertness to negative emotional information was closely related to the automated processing of their susceptibility [38, 40]. The correlation of attentional bias to negative emotional information and social anxiety severity would provide theoretical support for subsequent intervention training.

Study 2 showed that the high social anxiety group responded to negative emotional information faster than the low social anxiety group and the attentional bias scores of the high social anxiety group were significantly higher than those of the low social anxiety group. Study 2 demonstrated that the individuals with high social anxiety showed attentional bias to negative emotional information. During the attentional processing stage, negative and neutral stimuli often compete for the order in which they are processed. Among them, negative stimuli are prioritized for processing by individuals with social anxiety [38]. The formed negative attentional bias eventually produces anxiety [41].

The individuals with social anxiety had attentional hypervigilance to negative information with a presentation time less than 500 ms, while they tended to avoid negative information with the presentation time more than 500 ms [42]. It indicated that individuals with social anxiety tend to maintain strict vigilance against the negative information in the early stages of the cognitive processing and avoid negative information in the later stages [42]. That probably attentional hypervigilance occurs in early stages of attention process. The ABM intervenes the attentional bias to negative information in the early stages of attentional processing [19]. Finally, based on the conclusions of Study 1, Study 2 investigated the intervention effect of ABM on social anxiety. The results showed that attentional bias scores of the ABM group were significantly lower than those of the ACC group after intervention. In the ABM group, the attentional bias scores of the posttest were significantly lower than those of the pretest, indicating that ABM can reduce the attentional bias of individuals with social anxiety to negative emotional information. ABM training effectively reduced the level of social anxiety, and the ABM group showed a better intervention effect than the ACC group [16, 39]. Cognitive-behavioral theory suggests that attention processing plays a key role in the pathogenesis and maintenance of anxiety disorders [43, 44]. Timely intervention of such attentional bias could reduce individual social avoidance behavior and prevent the maintenance of anxiety [45].

Regarding the differing results of previous studies, as mentioned above, the settings of experimental materials, training environment and training time may affect the results. First, considering the individual’s recognition ability of emotional faces in the same cultural background during the training process and diversity of negative emotional faces in the real interpersonal communication environment, to improve the ecological validity of the experimental materials, emotional face pictures from CFAPS were used in the study. Second, participants might take the training task less seriously at home than in the laboratory setting. Other important differences between the laboratory and home environments were stress and arousal levels. The home environment might not evoke enough arousal to promote ABM training [46]. Therefore, considering the influence of the training environment, ABM in the laboratory setting was used in this study. Finally, considering that the inconsistent settings of training time interval and training times might also have a greater impact on the intervention effect, the study combined the problem of replicability and standardization of settings and extended intervention times while referring to the previous intervention experimental parameters. The number of training sessions significantly regulated posttest stress response [17]. The more training times there are, the greater the stress response. There might be a “dose‒response” relationship in the study of ABM; that is, the more intervention doses were activated pertinently, the stronger the observation results became (gradient criterion) [47]. More training was related to the improvement of anxiety symptoms.

This study first verified the correlation of attentional bias and social anxiety severity and then carried out training for attentional bias on this basis, which had a good theoretical basis. Previous studies failed to carry out research on the characteristics of attentional bias. According to the relevant viewpoints of information processing theory [38], through ABM, individuals with social anxiety can always track neutral stimuli, thereby reducing their attention to negative stimuli. In the posttest stage, social anxiety levels and attentional bias scores of individuals with social anxiety decreased. The reduction in negative attention might be the reason for reduction in social anxiety levels. In addition, ABM might reduce emotional vulnerability of participants in real-life social encounters [48], which would help alleviate anxiety symptoms of individuals with social anxiety. The research is carried out in the current social environment, which is different from the past. ABM still has a positive effect on improving the level of social anxiety, enriching the empirical research in the field of social anxiety intervention, and supports the promotion and application of ABM.

This study has limitations. First, the intervention time in this study was 10 days, and the immediate effect after intervention was significant. It is necessary to increase the follow-up time to evaluate the maintenance duration of the intervention effect. Second, the training paradigm of ABM can consider introducing virtual reality technology to explore ways to increase its interest and effectiveness so that individuals can have a better experience and participation in the process of intervention treatment [39, 49]. Nevertheless, this study established an effective intervention model for social anxiety that can be popularized and used in college mental health work. ABM training has the characteristics of being short term and timely, having no side effects, and being easy to operate. It improves the foresight and accuracy of college mental health work to solve practical work difficulties.

In sum, under conditions of this study, we found that ABM can reduce attentional bias of college students with social anxiety to negative emotional information and effectively improve their social anxiety levels.

### Supplementary Information

Supplementary Material 1.

## Data Availability

All data generated or analysed during this study are included in the supplementary information files.
